# Decentralized subject recruitment for a prospective community surveillance system: The influence of social determinants of health on inclusion of minorities in research

**DOI:** 10.1017/cts.2025.18

**Published:** 2025-03-27

**Authors:** Paul Takahashi, Chung-Il Wi, Robert Pignolo, Wendelyn Bosch, Katherine King, Euijung Ryu, Traci Natoli, Kathy Ihrke, Matthew Spiten, Lisa Speiser, Brandon Hidaka, Young Juhn

**Affiliations:** 1 Geriatrics and Palliative Care, Mayo Clinic Rochester, Division of Primary Care Internal Medicine, Rochester, MN, USA; 2 Mayo Clinic Rochester, Division of Community Pediatrics and Adolescent Medicine, Rochester, MN, USA; 3 Mayo Clinic Rochester, Divisons of Hospital Internal Medicine and Endocrinology, Rochester, MN, USA; 4 Mayo Clinic Florida, Division of Infectious Disease, Jacksonville, FL, USA; 5 Mayo Clinic Rochester, Department of Quantitative Health Sciences, Rochester, MN, USA; 6 Mayo Clinic Arizona, Division of Infectious Disease, Scottsdale, AZ, USA; 7 Department of Family Medicine, Mayo Clinic Health System, Eau Claire, WI, USA

**Keywords:** Decentralized, recruitment, cohort study, disparities, social determinants of health

## Abstract

**Background/Objective:**

Decentralized research has many advantages; however, little is known about the representativeness of a source population in decentralized studies. We recruited participants aged 18-64 years from four states from June to December 2022 for a prospective cohort study to assess viral epidemiology. Our aim was to determine the association between age, gender, race/ethnicity, rurality, and socioeconomic status (SES) on study participation in a decentralized prospective cohort study.

**Methods:**

We consented 9,286 participants from 231,099 (4.0%) adults with the mean age of 45.6 years (±12.0). We used an electronic decentralized approach for recruitment. Consented participants were more likely to be non-Hispanic White, female, older, urban residents, have more health conditions, and possessed higher socioeconomic status (SES) compared to those non-consented.

**Results:**

We observed an interaction between SES and race-ethnicity on the odds of consent (*P* = 0.006). Specifically, SES did not affect non-Hispanic white participation rates(OR 1.24 95% CI 1.16 – 1.32] for the highest SES quartile compared to those with the lowest SES quartile) as much as it did participants combined across the other races (OR 1.73; 95% CI 1.45 – 2.98])

**Conclusion:**

The relationship between SES and consent rates might be disproportionately greater in historically disadvantaged groups, compared to non-Hispanic White. It suggests that instead of focusing on enrollment of specific minority groups in research, there is value in future research exploring and addressing the diversity of barriers to trials within minority groups. Our study highlights that decentralized studies need to address social determinants of health, especially in under-resourced populations.

## Introduction

Poor recruitment is a primary reason for discontinuation of clinical trials, a major impediment to biomedical research [4]. There is no clear effective evidence-based recruitment strategy for prospective trials [5]. Investigators often utilize traditional recruitment approaches like direct recruitment in the hospital or clinic, advertisements, flyers, or paper mailing. These techniques often face the discussed barriers and are time-intensive. Telephone reminders do enhance recruitment rates of traditional methods [5]. Successful recruiting must be efficient, ethical, and effective at enrolling a representative sample from the population [1,2,3]. The Special Population Program Work Group of the Center for Translational Science Activities consortium recently provided a framework for addressing the barriers hindering recruitment from racial/ethnic minorities, people living in poverty, and participants living in rural areas [1]. These barriers include distrust of the medical system, limited time, and transportation issues [2,3]. Decentralized research has emerged as a potentially superior recruitment method.

In decentralized research, the patient remains at home throughout the study while the investigators work remotely. Decentralized research addresses some concerns regarding efficiency and safety [6]. Investigators could engage potential participants who have been excluded from research because of geography, transportation, or other barriers. Patients who live in rural areas often have limited access to clinical research because of the vast geographic dispersion of rural populations and their distance to research centers [7]. Decentralized research can accelerate recruitment of rare diseases [8] as this methodology typically uses the electronic health record (EHR) to enhance participant (such as rare disease) identification and assist with recruitment [9]. Traditional recruitment methods also utilize the EHR, however, decentralized research may more robustly leverage portal or other virtual tools integrated with EHRs enabling remote communication, consent, and measurement compared to a traditional study primarily relying on in-person interaction on site. The information from the EHR can quickly exclude participants who do not meet inclusion criteria like age, living proximity, or certain comorbid health conditions and can alert research teams about potential participants [10,11] There is an opportunity to proactively engage under-resourced groups and reduce barriers such as implicit bias by offering the study to all participants [12,13].

It is not clear if decentralized recruitment improves diverse representation because racial/ethnic minorities and under-resourced populations have systematic differences including lack of internet access (digital divide), ability to navigate a patient portal, other desired resources (e.g., support for performing study procedures at work) or even patient’s preferences and values [14]. Little is known about the role of SES, race/ethnicity, and residential settings in study participation using decentralized research. Specifically, it is poorly understood the extent to which SES, as a key element of social determinants of health (SDH), accounts for the impact of race/ethnicity and rurality on study participation as barriers to inclusion of special populations. Our primary aim is to compare the characteristics of participants who consented to study participation using decentralized research with those who did not consent (hereafter non-consented) in adults ages 18–64 years residing in our practice across the Midwest, Arizona, and Florida.

## Methods

### Study Setting and Design

The present investigation is an analysis of an ongoing large decentralized prospective case-cohort study designed to measure the incidence of respiratory syncytial virus (RSV) in adults 18 to 64. We recruited and followed patients enrolled in primary care at one of four Mayo Clinic campuses. We initiated the study in June 2022 and completed enrollment in December 2022. We conducted the study within the three geographic regions and four states in the United States as follows: upper Midwest (Mayo Clinic Rochester and Mayo Clinic Health System [MCHS] in Minnesota and Wisconsin), Mayo Clinic Florida and Mayo Clinic Arizona. Mayo Clinic campuses in Rochester, MN, Scottsdale, AZ, and Jacksonville, FL represent academic practices while MCHS is large set of community-based practices with 16 community hospitals and 53 clinics staffed by 1,000 clinicians across MN and WI, in primarily rural settings [15]. This represents a unique aspect of Mayo Clinic practices across 4 geographic regions under one institution which provides an opportunity for conducting a decentralized study recruiting participants across different regions such as this. The Mayo Clinic Institutional Review Board reviewed and approved the study. The study was conducted within the framework of the Declaration of Helsinki [16]. We reported our findings within the STROBE guidelines [17]. (Supplemental table one)

#### Technology-enabled subject recruitment system (TESRS)

We utilized technology-enabled subject recruitment system (TESRS) for efficient large decentralized subject recruitment, which was recently reported in detail [18]. Briefly, our team generated potential participants’ lists from the EHR and randomized the list for batch enrollment by each of the four study sites. We initiated TESRS including determining availability of email address in the patient record (to determine electronic vs. postal invitation) from the EHRs within the study sites. The invitation connected interested participants to a short online survey to determine eligibility. Their information was interfaced with Participant Tracking System (PTrax) of Mayo Clinic, a clinical trials management system that digitally consents participants. Study coordinators aided participants as an option for patients who encountered difficulty using the electronic consent and enrollment process.

### Participants

We invited adults aged 18–64 years with a listed Mayo Clinic primary care clinician and who had received medical care at a Mayo Clinic campus within 3 years prior to the study index date. For participants receiving medical care in MN, we required medical record research authorization in accordance with state statutes and confidentiality laws [19]. Participants receiving medical care in FL, AZ, and WI were not subject to research authorization. The study involved collecting biospecimens at home by a courier service; therefore, potential participants had to live within the catchment area of a medical courier service (MedSpeed LLC. Elmhurst, IL.). The courier service used regional hubs with a 30-mile radius which covered more than 90% of the potential participants. After establishing a population-based sampling frame, we executed a random sampling frame and recruited study subjects from the sampling frame accordingly.

### Recruitment

We recruited and consented to the participants remotely. Participants received either an electronic or mailed invitation depending on their EHR-listed preferred contact method. Emailed invitation letters provided a detailed description of the study and included an electronic pre-screening survey which contained the inclusion/exclusion criteria through TESRS which was signed electronically via PTrax. We mailed the invitation letter along with a pre-paid envelope to those who opted out of electronic options. Once we received notification from interested and eligible participants, we contacted them over the phone via an IRB-approved phone script to confirm inclusion criteria and enroll if eligible for the study. Once the participant met the inclusion criteria, we consented to the participants using one of the following: remote electronic consenting and DocuSign technology to collect signatures or a mailed consent.

### Primary outcomes

The primary outcome was consent status affirming a desire to participate in the study. Participants were considered to consent if they signed the consent form either electronically or via paper. The non-consented group included those potential participants whom we contacted but did not consent to the study. Other outcomes included the characteristics of those who consented to each site. We also reported the number of participants consented per month overall and per site of recruitment.

### Predictor Variables

We collected demographic, socioeconomic, geographic, and medical comorbidity characteristics from the EHR including the following: demographic information, billing information using International Statistical Classification of Diseases and Related Health Problems, 10th revision (ICD-10), and SES. We categorized age at the time of enrollment using categories 18 – 49, 50 – 59, and 60 – 64, and reported age as a continuous variable. We used patient-reported gender as male, female, or missing. We reported self-described race and ethnicity as African American, Asian, American Indian/Alaska Native, Native Hawaiian/ Pacific Islander, Hispanic, Latino, non-Hispanic White, and unknown. Because of the smaller number of participants in each group, we also categorized non-Hispanic White and other groups.

For socioeconomic status, we used a validated, standardized, and objective individual-level SES measure, the individual housing-based SES index, and the HOUsing-based SocioEconomic Status measure (HOUSES) index. The HOUSES index is a validated, standardized, and objective patient-level SES measure. It is based on participant’s address in the EHR and its associated publically available housing data from the Office of the County. The HOUSES index is based on four real property variables: the assessor’s value, square footage of the housing unit, number of bedrooms, and number of bathrooms in the individual home. HOUSES index is available for the entire 50 states and has been used for numerous epidemiological studies predicting more than 50 outcomes in adults and children reported in more than 30 publications [20–24]. A higher HOUSES index score indicates a higher SES level [22]. We reported HOUSES level based upon quartiles. We classified participants according to their address as living in a rural area or an urban area based on the US Census Bureau’s rural and urban classification [25]. For geographical predictors, we reported the panel group from the midwest (MN, WI), FL, and AZ.

For medical comobidity predictors, we used the Charlson Comobidity Index to summerize comorbid health burden by counting the number of chronic health conditions [26,27] We reported the sum count of these illnesses and categorized the sum of chronic conditions into three levels: zero conditions, one condition, or two or greater conditions.

### Statistical analysis

For the primary analysis, we reported differences in predictors between those potential participants who had consented and non-consented. We used the Chi Square test for categorical variables and Kruskal–Wallis test for continuous variables to calculate p-values. We considered a *p* value less than 0.05 significant. We calculated logistic regression to investigate the association of demographic factors with consent status. We reported odds ratios (OR) with 95% confidence intervals unadjusted and also adjusted for age, gender, and HOUSES index to determine association of consent status by race/ethnicity, regional status, and sum of chronic conditions. We assessed the interaction between SES and race/ethnicity and between SES and residential settings (rural vs. urban) on study participation. We reported stratified race/ethnicity and residential-specific odds ratios for HOUSES quartiles. We used the reference as the first quartile of the HOUSES index (lowest SES). We reported monthly recruitment efforts by geographic region of Midwest, FL, and AZ.

## Results

### Subject characteristics for consented vs. non-consented participants

We contacted a total of 231,099 patients as potentially eligible for the study (i.e., sampling frame). Ineligible patients were not included in either consented or non-consented group. Those excluded because of catchment were similar in age and gender and were higher proportion of non-Hispanic White (91% versus 82% in sample). We consented to 9,286 participants (4.0%) of this population in all four states over a six-month period from June 2022 to December 2022. The mean age of those consented was 45.6 years (± 12.0) compared to 42.1 years (± 13.8) of those non-consented. Consented participants were more likely non-Hispanic White (89.6% vs. 82.1% in non-consented) and female (71.4% vs. 54.4% in non-consented). We found lower participation in participants with the lowest SES as measured by HOUSES quartile (16% vs. 20% in non-consented) and rural residents (21.2% vs. 22.6% in non-consented). We also found that of consented participants, 12.5% had 2 or more comorbid health conditions compared to 9.6% in those non-consented (*p* < 0.001) (Table 1)

**Table 1.** Consented versus non-consented participants in 231,099 adults

	Consented (*N* = 9,286)	Not Consented (*N* = 221,813)	*p* value
Age Categories			< 0.001
18 – 49	5,367 (57.8%)	142,912 (64.4%)	
50 – 59	2,473 (26.6%)	49,137 (22.2%)	
60 – 64	1,446 (15.6%)	29,764 (13.4%)	
Age			< 0.001
Mean (SD)	45.6 (12.0)	42.1 (13.8)	
Range	18 – 64	17 – 64	
Gender			< 0.001
N-Miss	0	20	
Male	2,655 (28.6%)	101,124 (45.6%)	
Female	6,631 (71.4%)	120,669 (54.4%)	
Race			< 0.001
Non-Hispanic White	8, 313 (89.5%)	181,837 (82.0%)	
African American	193 (2.1%)	9,982 (4.5%)	
Asian	274 (3.0%)	9,845 (4.4%)	
Native Hawaiian/Pacific Islander/American Indian/Native Alaskan	45 (0.5%)	1056 (0.5%)	
Hispanic or Latino	338 (3.6%)	13,145 (5.9%)	
Unknown	123 (1.3%)	5,948 (2.7%)	
Rurality			< 0.001
N-Miss	223	6556	
Rural	1,917 (21.2%)	48,741 (22.6%)	
Urban	7,146 (78.8%)	166,516 (77.4%)	
HOUSES			< 0.001
N-Miss	219	6554	
1	1,451 (16.0%)	42,793 (19.9%)	
2	1,649 (18.2%)	41,560 (19.3%)	
3	2,413 (26.6%)	53,071 (24.7%)	
4	3,554 (39.2%)	77,835 (36.2%)	
Panel			< 0.001
Midwest	6,615 (71.2%)	165,647 (74.7%)	
Florida	1,425 (15.3%)	29,396 (13.3%)	
Arizona	1,246 (13.4%)	26,770 (12.1%)	
Sum of Diseases			< 0.001
0	5,758 (62.0%)	156,569 (70.6%)	
1	2,370 (25.5%)	43,851 (19.8%)	
2 or more	1,158 (12.5%)	21,393 (9.6%)	

In unadjusted analysis, female gender was associated with consenting to participate: OR 2.09 (95% CI 2.00 – 2.19) compared to males. Compared to ages 18 – 49 years, we found age category of potential participants was also associated with consenting with an odds ratio of 1.34 (95% CI 1.28 – 1.41) in the 50 – 59 category and 1.29 (95% CI 1.22 – 1.37) in the 60-64 category (Table 2). After adjustment for age, gender, and HOUSES index quartile, potential participants living in urban areas were more likely to consent with an OR 1.14 (95% CI 1.08 – 1.20) compared to those living in a rural area. Compared to non-Hispanic White participants, consent was less likely among African-American participants OR 0.47 (95% CI 0.40 – 0.54), Asian participants OR 0.61 (95% CI 0.54 – 0.69), and Hispanic or Latino ethnicity participants OR 0.60 (95% 0.53 – 0.67). Consent was not significantly lower among American Indian/Native Hawaiian/Pacific Islander/Alaskan Native participants (OR 0.93, 95% CI: 0.68 to 1.24). (Table 2).

**Table 2.** Unadjusted and age/gender/HOUSES adjusted odds ratios for odds of consenting by sociodemographics with 95% confidence intervals

	Unadjusted				Age, Gender, HOUSES adjusted			
	OR	Lower CI	Upper CI	*P* value	OR	Lower CI	Upper CI	*P* value
Age Categories 18 – 49	1	ref	ref					
Age Categories 50 – 59	1.340	1.276	1.407	< 0.001	–	–	–	–
Age Categories 60 – 64	1.294	1.219	1.372	< 0.001	–	–	–	–
Gender Male	1	ref	ref					
Gender Female	2.093	2.000	2.191	< 0.001			--	
HOUSES Quartile 1	1	ref	ref		–	–	–	
HOUSES Quartile 2	1.170	1.089	1.257	< 0.001	–	–	–	
HOUSES Quartile 3	1.341	1.255	1.433	< 0.001	–	–	–	
HOUSES Quartile 4	1.347	1.266	1.433	< 0.001	–	–	–	
Race Non-Hispanic White	1	ref	ref		1	ref	ref	
Race African American	0.423	0.365	0.487	< 0.001	0.467	0.402	0.539	< 0.001
Race Asian	0.609	0.538	0.687	< 0.001	0.609	0.537	0.687	< 0.001
Race American Indian/Alaskan Native/Native Hawaiian/Pacific Islander	0.932	0.681	1.241	0.645	0.969	0.705	1.296	0.840
Race Hispanic or Latino	0.562	0.503	0.627	< 0.001	0.598	0.534	0.668	<0.001
Race Unknown	0.452	0.376	0.539	< 0.001	0.485	0.401	0.581	<0.001
Rurality Rural	1	ref	ref		1	ref	ref	
Rurality Urban	1.091	1.037	1.149	< 0.001	1.139	1.082	1.200	<0.001
Region Midwest	1	ref	ref		1	ref	ref	
Region Florida	1.214	1.144	1.287	< 0.001	1.043	0.981	1.107	0.176
Region Arizona	1.166	1.095	1.239	< 0.001	0.968	0.907	1.032	0.324
Sum of Diseases Zero	1	ref	ref		1	ref	ref	
Sum of Diseases One	1.470	1.399	1.543	< 0.001	1.336	1.270	1.405	<0.001
Sum of Diseases Two or more	1.472	1.379	1.570	< 0.001	1.264	1.179	1.353	<0.001

*Note:* OR = Odds ratio; CI = confidence interval.

### Impact of SES on inclusion of racial/ethnic minorities and rural population

**Figure 1.**
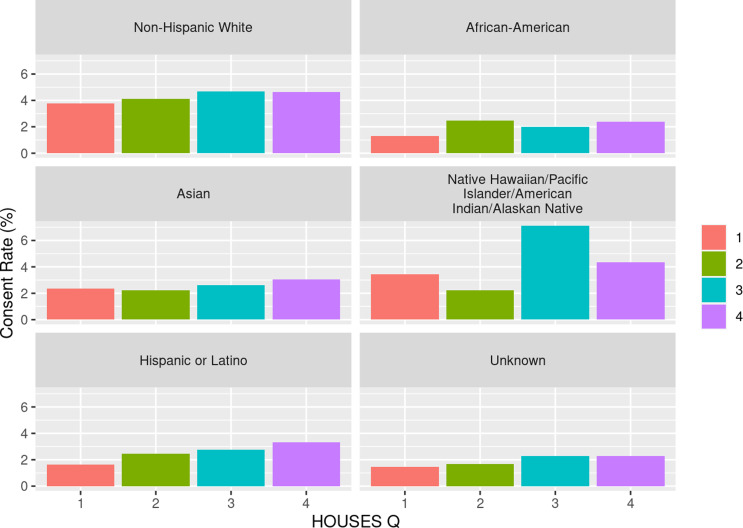
Consent rate (percentage) by HOUSES within self-reported race and ethnicity. HOUSES quartile from 1 to 4 with 1 having the lowest socioeconomic status and 4th quartile having the highest socioeconomic status.

**Figure 2.**
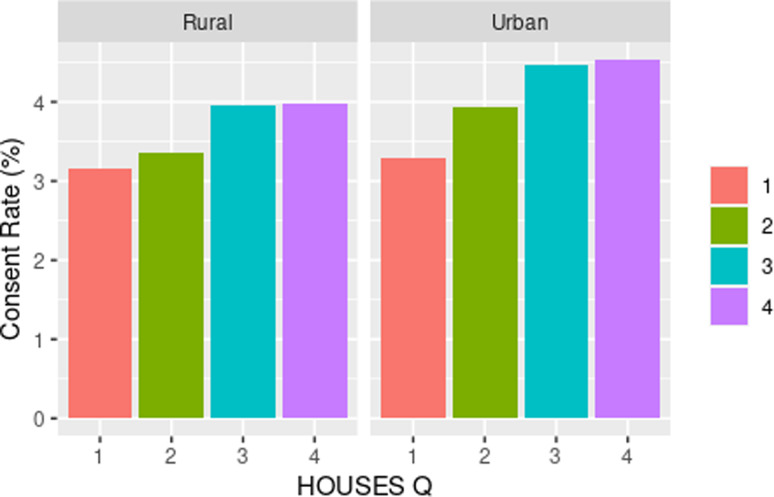
Consent rate (percentage) by HOUSES within rurality. HOUSES quartile from 1 to 4 with 1 having the lowest socioeconomic status and 4th quartile having the highest socioeconomic status.

As shown in Table 3, we observed a dose-response relationship between SES and consent rate with higher SES associated with higher response rate among all racial groups. We assessed the impact of SES as a key element of SDH on inclusion of race/ethnicity in study participation as shown in Fig. 1 and with residential settings in study participation as shown in Fig. 2. As shown in both figures, SES impacts participation of all races/ethnic groups and patients with different residential settings in our research. The interaction of SES and the six race/ethnicity groups was not significant (*P* = 0.108); however, this could be due to the small sample sizes in some groups. (Table 3) Importantly, when dichotomizing race-ethnicity into non-Hispanic White versus other race/ethnic group, we did see a significant interaction with SES (*p* = 0.006). The effect of SES was much more dramatic in the minority racial and ethnic groups compared to the non-Hispanic White group. In the highest HOUSES quartile for non-Hispanic White participants, the OR was 1.24 (95% CI 1.16 – 1.32) compared to the lowest HOUSES quartile. In all other racial groups and Hispanic ethnicity, the OR for consent in the highest HOUSES was 1.73 (95% CI 1.45-2.08) (Supplemental table one) (supplemental figure one). There was no interaction between rurality and HOUSES (*P* = 0.725). In the rural population, the odds of consenting were higher in the highest HOUSES quartile with OR 1.27 (95% CI 1.07 – 1.50) compared to lowest quartile, (see Table 3) and a similar effect was seen in the urban population. In the urban population, the odds of consenting were highest in the highest quartile of 1.39 with 95% CI of 1.30 – 1.49. There was no interaction between rurality and HOUSES.

**Table 3.** Race and rurality specific odds ratios for odds of consenting by HOUSES with 95% confidence intervals

		OR	Lower CI	Upper CI	*P* value	Interaction *P* value
Race						0.108
African American	HOUSES Quartile 1	1	ref	ref		
	HOUSES Quartile 2	1.905	1.250	2.905	0.003	
	HOUSES Quartile 3	1.506	0.989	2.295	0.057	
	HOUSES Quartile 4	1.827	1.228	2.716	0.003	
Non-Hispanic White	HOUSES Quartile 1	1	ref	ref		
	HOUSES Quartile 2	1.095	1.015	1.183	0.020	
	HOUSES Quartile 3	1.253	1.168	1.345	<0.001	
	HOUSES Quartile 4	1.236	1.157	1.321	<0.001	
Asian	HOUSES Quartile 1	1	ref	ref		
	HOUSES Quartile 2	0.945	0.594	1.505	0.813	
	HOUSES Quartile 3	1.108	0.747	1.642	0.611	
	HOUSES Quartile 4	1.302	0.922	1.837	0.134	
American Indian/Alaskan Native/Native Hawaiian/ Pacific Islander	HOUSES Quartile 1	1	ref	ref		
	HOUSES Quartile 2	0.631	0.213	1.874	0.407	
	HOUSES Quartile 3	2.136	0.950	4.802	0.066	
	HOUSES Quartile 4	1.273	0.549	2.950	0.574	
Hispanic or Latino	HOUSES Quartile 1	1	ref	ref		
	HOUSES Quartile 2	1.504	1.069	2.114	0.019	
	HOUSES Quartile 3	1.700	1.218	2.373	0.002	
	HOUSES Quartile 4	2.059	1.504	2.818	< 0.001	
Unknown	HOUSES Quartile 1	1	ref	ref		
	HOUSES Quartile 2	1.141	0.599	2.173	0.689	
	HOUSES Quartile 3	1.555	0.886	2.730	0.124	
	HOUSES Quartile 4	1.565	0.934	2.620	0.089	
Rurality						0.725
Rural	HOUSES Quartile 1	1	ref	ref		
	HOUSES Quartile 2	1.064	0.875	1.293	0.536	
	HOUSES Quartile 3	1.266	1.059	1.513	0.010	
	HOUSES Quartile 4	1.270	1.073	1.502	0.006	
Urban	HOUSES Quartile 1	1	ref	ref		
	HOUSES Quartile 2	1.199	1.110	1.296	< 0.001	
	HOUSES Quartile 3	1.371	1.275	1.473	< 0.001	
	HOUSES Quartile 4	1.392	1.300	1.490	< 0.001	

*Note:* OR = Odds ratio; CI = confidence interval.

### Regional comparisons

Regional comparison showed that we recruited younger subjects in the Midwest with 64% of the cohort ages 18-49 years compared to 46% in FL and 38.8% in AZ (*p* value <0.001). We did not see a difference in gender. We did see that non-Hispanic Whites were 92.7% in the Midwest compared to 81.2% in FL and 82.7% in AZ. 25% of the participants lived in a rural area in the Midwest compared to 15.5% in FL and 7.3% in AZ (*p* < 0.001). We found that AZ had 15.0% of patients with 2 or more chronic illnesses compared to 12.9% in FL and 11.9% in the Midwest (*p* = 0.007). We did find that participants from both FL and AZ had higher SES, with both groups having over 50% in the highest HOUSES quartile compared to 34.3% in the Midwest (*p* < 0.001) (Table 4). Recruitment rates were highest in the earlier part of the study. (Supplemental figure two)

**Table 4.** Comparison of recruited participants by location

	Midwest (*N* = 6,616)	Florida (*N* = 1,424)	Arizona (*N* = 1,246)	*p* value
**Age Categories**				< 0.001
18 – 49	4,228 (63.9%)	655 (46.0%)	484 (38.8%)	
50 – 59	1,522 (23.0%)	475 (33.4%)	476 (38.2%)	
60 – 64	866 (13.1%)	294 (20.6%)	286 (23.0%)	
**Age**				< 0.001
Mean (SD)	44.1 (12.0)	48.9 (11.1)	50.2 (10.8)	
Range	18 - 64	18 - 64	19 - 64	
**Gender**				0.061
Male	1,849 (27.9%)	441 (31.0%)	365 (29.3%)	
Female	4,767 (72.1%)	983 (69.0%)	881 (70.7%)	
**Race**				< 0.001
Non-Hispanic White	6,132 (92.7%)	1,152 (80.9%)	1,029 (82.6%)	
African American	75 (1.1%)	86 (6.0%)	32 (2.6%)	
Asian	144 (2.2%)	74 (5.2%)	56 (4.5%)	
American Indian/Alaskan Native/ Native Hawaiian/Pacific Islander	28 (0.4%)	10 (0.7%)	7 (0.6%)	
Hispanic or Latino	162 (2.4%)	70 (4.9%)	106 (8.5%)	
Unknown	75 (1.1%)	32 (2.3%)	16 (1.3%)	
**Rurality**				< 0.001
N-Miss	166	21	36	
Rural	1,612 (25.0%)	217 (15.5%)	88 (7.3%)	
Urban	4,838 (75.0%)	1,186 (84.5%)	1,122 (92.7%)	
**HOUSES Quartile**				< 0.001
N-Miss	163	21	35	
1	1,190 (18.4%)	141 (10.0%)	120 (9.9%)	
2	1,342 (20.8%)	154 (11.0%)	153 (12.6%)	
3	1,710 (26.5%)	395 (28.2%)	308 (25.4%)	
4	2,211 (34.3%)	713 (50.8%)	630 (52.0%)	
**Sum of Diseases**				0.007
0	4,157 (62.8%)	847 (59.5%)	754 (60.5%)	
1	1,671 (25.3%)	394 (27.7%)	305 (24.5%)	
2 or more	788 (11.9%)	183 (12.9%)	187 (15.0%)	

## Discussion

In this study of 9,286 participants, we discovered novel findings using decentralized research as our primary method of recruiting diverse patients. We found that the characteristics of the consented cohort may be affected by the recruitment strategy. The representativeness of the study sample in our decentralized recruitment strategy appears to be impacted by race/ethnicity, residential settings, and SES of our study population. We observed higher SES, as a key element of SDH, increased consent rates. This higher SES increased consent rates in both urban and rural residents as well as all race/ethnic groups. While lower consent rates among minority populations and under-resourced populations are widely recognized, little is known about the interaction between SES and race/ethnicity. Our study results suggest that the effect of SES on consent rates was highest in the combined under-represented groups compared to those that self-identify as non-Hispanic White.

There is a scant number of decentralized studies that report factors associated with consent rates, specifically the role of SES and other SDH in inclusion of special populations such as under-represented populations, rural populations, and under-resourced populations. To our knowledge, this is the first study to assess the extent to which SES accounted for the impact of race/ethnicity and rurality on study participation in decentralized studies [1]. While there is no reason to believe decentralized studies are immune to selection bias, like traditional studies, our study results showed that decentralized recruitment strategy is potentially susceptible to selection bias and representativeness of the study sample for a source population. Specifically, the age of those who consented versus non-consented was slightly older by 3 years and were more than twice as likely to be female. In addition to demographic factors, geographic factors also played a role in recruiting a diverse group. For example, recruitment of under-represented groups was higher in Florida and Arizona with a 6-fold increase in African American recruitment in Florida. In Olmsted County, MN, the population reflects a percentage of 8% Black or African American [28], compared to 31% of Black or African American in Duval County, FL [29]. Recruiting from different regions of the country may better reflect the geographic and population representation, given the variability of RSV epidemiology (our study aim) by regions and population. These are important considerations using decentralized research.

Our findings of higher enrollment with higher SES using decentralized research have been seen with investigators using traditional approaches of recruitment. In the Mayo Clinic Biobank, investigators enrolled a larger proportion of individuals with higher SES as measured by higher education levels compared to the census population in the region [30]. In a pharmacogenomics study from the same region, the recruited population reported that 58% had a bachelor’s degree compared to 15% in the surrounding counties [31]. We were interested in assessing the impact of SES on inclusion of different racial and ethnic groups in research (i.e., interaction). Based on analysis for individual racial/ethnic group, we found that within the African American group, those in the highest SES quartile had 83% higher odds of consenting compared to the lowest quartile. In participants with Hispanic ethnic origin the highest SES had 2-fold increased odds of consenting, compared to those with the lowest SES. Thus, the group least likely to consent are those in the lowest SES group within the under-represented minority group. This is an important finding as research groups strive to understand populations at risk for both under-represented minorities and under-resourced populations. Importantly, based on our analysis for binary racial groups (non-Hispanic White vs. other under-represented groups), the impact of SES on study participation (consent rate) was significantly greater in under-represented populations than non-Hispanic White population. Specifically, the consent rate for participants in under-represented groups in the highest SES category was 73% higher than in the lowest SES category. This compares to only a 24% increased participation rate in the highest SES category compared to lowest category in non-Hispanic White population. Our study results offer an important insight into the potential role of SES as a key element of SDH as a potential factor underlying disparities in inclusion of under-represented groups. Addressing participants’ SDH will be a crucial factor for addressing such disparities because SES is defined as one’s ability to access desired resources [32]. In addition, our study results underscore the importance of exploring and addressing a broad range of barriers (e.g., SDH) to research within minority groups instead of solely focused on increasing participation among racial/ethnic patient groups. Previous methods of prospective recruitment have indicated bias in recruitment. Previous reviews of bias in clinical trials have suggested underrepresentation of females, Hispanics, American Indian, Alaskan natives, Asians, and Whites [15]. Investigators have found that using a patient portal for research recruitment improved recruitment of women using an electronic method [12]. Our study differs and adds additional information as we did not use the patient portal for communication. There has been great interest in using decentralized research to help reduce bias in recruiting participants [33]. Our findings suggest that despite the reduction in implicit bias in inviting under-represented groups to participate in research, we still found enrollment lower in underrepresented minority groups compared to non-Hispanic Whites. There remains work to be done with different strategies for recruitment of under-represented groups [34] and the use of decentralized research alone may be inadequate to ensure better representation. However, researchers should still consider the potential benefit of decentralized research for enrolling under-represented groups because of barriers to access to an urban research center [14].

Decentralized research has potential areas for growth and refinement as this is a newer method of research execution. Given the consumer-driven health care and its rapid change, health care systems and researchers face a situation where patients, as consumers request providers, consider their values and preferences in all health care decisions including designing and planning clinical and translational studies. With attention to a patient’s preferences and values as well as implicit bias by racism, one must recognize potential shortcomings of decentralized research. First, the clinical and research information is only as robust as the data within the electronic system. Some groups have voiced concerns about data quality as a concern with decentralized research [23,35]. In particular, if patients have less access to personal health records with a lower SES [36], there may be more risks for inadequate capture of health information and even causing machine learning model bias and further exacerbating health disparities [23]. For decentralized research using internet-based recruitment, there is lower internet availability in lower SES participants [37]. The digital divide may provide some explanation for the findings in our study. Lastly, access to medical providers may play a role in recruitment. There are differences in primary care access in rural versus urban communities which makes medical access challenging [38].

Our study has strengths which include the practical application and experience of using decentralized research in a group of over 230,000 potential participants. First, we applied an innovative digital tool, TESRS, which reduces the burden on study coordinators [18]. Second, although our study was performed by a single institution, we made an effort to include multiple geographic regions to capture diverse study populations with a unified EHR. Lastly, we avoided potential concerns about data and safety by having medical investigators at all sites. We also allowed mail options to account for those without access to digital devices. We recognize there are limitations with the study involving the demographics of the empaneled primary care population which over-represent non-Hispanic Whites compared to the national population [39]. We also recognize that the service radius of the courier service may underrepresent rural participants. Our results may not generalize to other health systems or countries because of unique data systems, privacy issues, or access to health care. Using decentralized research for a clinical trial longitudinally may differ from our results. There is the potential for misclassification of information on coding illnesses, inaccurate living situations, and bias on self-report of gender, race, or ethnicity. We do not believe these potential classification errors would differ systematically between consented and non-consented groups or between regions.

## Conclusion

Our decentralized recruitment strategy was an efficient method of recruitment and potentially broadened access to different populations of under-represented groups. However, patients who consented to decentralized research were older, non-Hispanic White, female, urban residents, and had higher SES compared to those who non-consented. We found that higher SES in all groups had higher consent rates with a particular attention to higher recruitment in African American and Hispanic populations. However, the effect of SES on consent rates was much more dramatic in racial/ethnic under-represented groups than non-Hispanic White. Addressing SDH might be a crucial step toward improving inclusion of special populations in research and intervention studies. In this endeavor, the HOUSES index, which efficiently identifies an under-resourced population with limited access to resources, can be a useful tool for accelerating such effort at a national level.

## Supporting information

Takahashi et al. supplementary material Takahashi et al. supplementary material

## Supplementary material

The supplementary material for this article can be found at https://doi.org/10.1017/cts.2025.18.
