# Supplementary material for: Decentralized subject recruitment for a prospective community surveillance system: The influence of social determinants of health on inclusion of minorities in research
Source: J Clin Transl Sci. 2025 Mar 27;9(1):e104. doi: 10.1017/cts.2025.18 (PMC12100557; doi:10.1017/cts.2025.18)

Supplemental Table One: Interaction of Race and Ethnicity and HOUSES Index Quartile

|  |  | **Odds ratio** | **95% CI** | **P Value** |
| --- | --- | --- | --- | --- |
| Non-Hispanic White | Houses Q2 | 1.095 | 1.015-1.183 | 0.02 |
|  | Houses Q3 | 1.253 | 1.168-1.345 | <0.001 |
|  | Houses Q4 | 1.236 | 1.157-1.321 | <0.001 |
| Other Race and Ethnicity | Houses Q2 | 1.369 | 1.109-1.691 | 0.004 |
|  | Houses Q3 | 1.528 | 1.256-1.860 | <0.001 |
|  | Houses Q4 | 1.733 | 1.448-2.975 | <0.001 |

Supplemental Figure One Consent Rate: (percentage) by HOUSES within race and ethnicity


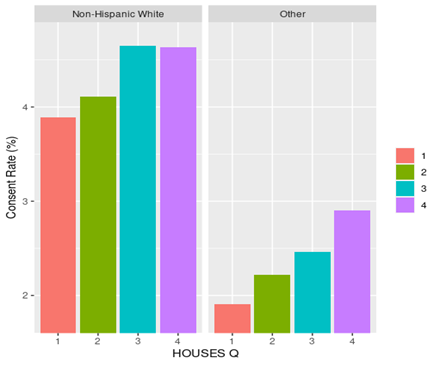


Legend: Houses Quartile from 1 to 4 with 1 having the lowest socioeconomic status and 4^th^ quartile having the highest socioeconomic status.

Supplemental Figure 2: Consent Recruitment Charts for Midwest, Florida and Arizona


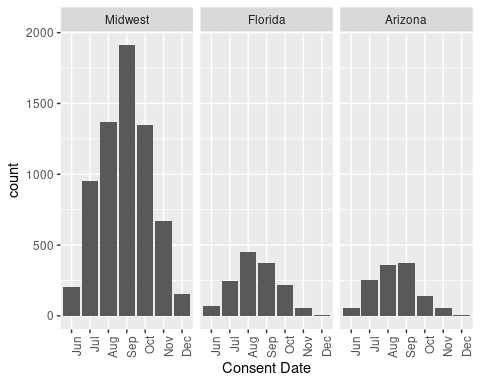

Supplement: Takahashi et al. supplementary material [file S2059866125000184sup001.docx]
